# CaMKII regulation of cardiac ryanodine receptors and inositol triphosphate receptors

**DOI:** 10.3389/fphar.2014.00101

**Published:** 2014-05-08

**Authors:** Emmanuel Camors, Héctor H. Valdivia

**Affiliations:** Division of Cardiovascular Medicine, Department of Internal Medicine, Center for Arrhythmia Research, University of Michigan, Ann ArborMI, USA

**Keywords:** ryanodine receptors, inositol triphosphate receptor, CaMKII, sarcoplasmic reticulum, Ca^2+^ leak, myocardium

## Abstract

Ryanodine receptors (RyRs) and inositol triphosphate receptors (InsP_3_Rs) are structurally related intracellular calcium release channels that participate in multiple primary or secondary amplified Ca^2+^ signals, triggering muscle contraction and oscillatory Ca^2+^ waves, or activating transcription factors. In the heart, RyRs play an indisputable role in the process of excitation–contraction coupling as the main pathway for Ca^2+^ release from sarcoplasmic reticulum (SR), and a less prominent role in the process of excitation–transcription coupling. Conversely, InsP_3_Rs are believed to contribute in subtle ways, only, to contraction of the heart, and in more important ways to regulation of transcription factors. Because uncontrolled activity of either RyRs or InsP_3_Rs may elicit life-threatening arrhythmogenic and/or remodeling Ca^2+^ signals, regulation of their activity is of paramount importance for normal cardiac function. Due to their structural similarity, many regulatory factors, accessory proteins, and post-translational processes are equivalent for RyRs and InsP_3_Rs. Here we discuss regulation of RyRs and InsP_3_Rs by CaMKII phosphorylation, but touch on other kinases whenever appropriate. CaMKII is emerging as a powerful modulator of RyR and InsP_3_R activity but interestingly, some of the complexities and controversies surrounding phosphorylation of RyRs also apply to InsP_3_Rs, and a clear-cut effect of CaMKII on either channel eludes investigators for now. Nevertheless, some effects of CaMKII on global cellular activity, such as SR Ca^2+^ leak or force-frequency potentiation, appear clear now, and this constrains the limits of the controversies and permits a more tractable approach to elucidate the effects of phosphorylation at the single channel level.

## CaMKII REGULATION OF CARDIAC RYANODINE RECEPTORS

### GENERAL CONSIDERATIONS

Ryanodine receptors (RyRs) are the calcium release channels of sarcoplasmic reticulum (SR) that provide the majority of the calcium ions (Ca^2+^) that are needed for contraction of cardiac and skeletal muscle. In the heart, these intracellular Ca^2+^ release channels are essential for normal cell development and indispensable for life, as demonstrated by the fact that genetic ablation of *RYR2*, the gene encoding for the cardiac isoform of the RyR channel (RyR2), causes death at early embryonic stage in mice (Takeshima et al., 1998). In humans, single amino acid mutations in RyR2 lead to life-threatening cardiac arrhythmias, also as demonstrated by the ~160 mutations that have been linked so far to catecholaminergic polymorphic ventricular tachycardia (CPVT; Priori and Chen, 2011). Thus, in their intracellular environment, RyR2 channels must be finely regulated so that their output signal (Ca^2+^) may induce finely graded cell contraction without igniting cellular processes that may lead to aberrant electrical activity or pathological cellular remodeling. This review will concentrate on regulation of RyR2 by *phosphorylation*, a common post-translational process that confers dynamic functional modulation to a myriad of ion channels and transporters. In the majority of cases, phosphorylation of ion channels or transporters provides an additional layer of regulation without actually being the effector or trigger of activity, i.e., in the case of RyR channels, phosphorylation *modulates* the effect of Ca^2+^ on cardiac RyR (RyR2) or depolarization on skeletal RyR (RyR1) without having the inherent ability to open or close the channel *per se*. In a useful analogy, phosphorylation and Ca^2+^/depolarization would be similar to the “volume” and “on/off” switches, respectively, of the radio in which RyR songs are played. However, as we will see on several sections of this review, this mode of regulation is so controversial that a definitive role for RyR2 phosphorylation has not been reached yet, despite the paramount importance of this process in physiological and pathophysiological conditions. RyR2 may be phosphorylated *in vitro* and *in vivo* by several kinases, but here we will concentrate on CaMKIIδ (the most abundant CaMKII expressed in the heart and referred to here simply as “CaMKII”), and incorporate protein kinase A (PKA) whenever relevant, as these two kinases share some common transduction pathways, bear the most relevance in cardiac diseases and have been studied the most. Similarly, at least two splice variants of CaMKIIδ are expressed in cardiomyocytes, CaMKIIδ_C_ (cytosolic) and CaMKIIδ_B_ (nuclear), and we will identify their differential effect whenever appropriate.

### RyR2 CHANNEL PROTEIN: COMPLEX STRUCTURE AND MULTIPLE PHOSPHORYLATION SITES

The RyR2 channel is a homotetrameric protein of ~2 million Da that is often complexed with several accessory proteins (see below), forming an intricate multi-protein array (Marx et al., 2000; Bers, 2004; Valdivia, 2013). The RyR2 monomer (almost 5,000 amino acids) is organized as a series of discrete domains, with the carboxy-terminal segment crossing the SR membrane as few as four and as many as ten times (depending on the model) and forming the Ca^2+^-permeable pore, whereas the bulk of the protein (~90%) protrudes into the cytosol to bridge a ~15–20 nm gap between the SR and t-tubule membranes (Hamilton and Serysheva, 2009; Capes et al., 2011; Van Petegem, 2012). The cytosolic portion of the channel contains multiple regulatory domains, most prominently Ca^2+^ activation and inactivation sites, but also binding sites for energy sensors such as nucleotides (ATP, ADP, and AMP) and inorganic phosphate, metabolites such as pyruvate, fatty acids and polyamines, and ions such as Mg^2+^, H^+^, and Cl^-^ (Zucchi and Ronca-Testoni, 1997; Fill and Copello, 2002; Meissner, 2004). The cytoplasmic site harbors also multiple phosphorylation epitopes (see below). Therefore, the RyR2 channel may act as a molecular switchboard that integrates a multitude of cytosolic signals such as dynamic and steady Ca^2+^ fluctuations, oxidation, metabolic states, β- adrenergic stimulation (phosphorylation), etc. and transduces these cytosolic signals to the channel pore to release appropriate amounts of Ca^2+^. Furthermore, Ca^2+^ release is critically influenced by *luminal* (intra-SR) factors such as Ca^2+^ content and protein interactions, thus conferring RyR2 channels an additional role as integrative switch-valves that restrict luminal Ca^2+^ overload, e.g., during sympathetic stimulation. Most of the signal-decoding structures are integral domains of the RyR2 protein, but as if this huge structural assembly were not sufficiently complex, RyR2 channels are also capable of protein–protein interactions that allow them to bind, in some cases steadily and in other cases in a time- and Ca^2+^-dependent manner, to smaller and independently regulated accessory proteins that add another layer of versatility (and complexity) to regulation of Ca^2+^ release *in vivo*. The best known RyR2-interacting proteins are calmodulin (CaM), which tonically inhibits Ca^2+^ release (Balshaw et al., 2002; Yang et al., 2014) FKBP12.6, which stabilizes RyR2 closures (Marx et al., 2000; Kushnir and Marks, 2010; but see also Timerman et al., 1996; Xiao et al., 2007), sorcin, which inhibits Ca^2+^ release in a Ca^2+^-dependent manner (Farrell et al., 2003), and the ternary complex triadin-junctin-calsequestrin, which “senses” luminal Ca^2+^ content and modulates RyR2 activity, probably by acting as direct channel ligands (Gyorke et al., 2004). More recently, RyR2 has been found to hold anchoring sites for PKA, protein phosphatase 1 (PP1) and 2A (PP1 and PP2A), phosphodiesterase 4D3 (PDE4D3) and CaMKII (Marx et al., 2000; Currie et al., 2004; Lehnart et al., 2005), underscoring the importance of RyR2 regulation by *phosphorylation*.

### RyR2 AS SUBSTRATE FOR CaMKII AND PKA

The multi-protein complex described above that includes kinases and phosphatases associated to the RyR2 strongly suggest that this channel is an avid target of downstream signaling effectors of the β-adrenergic system. PKA is a classical effector of the β-adrenergic pathway and, although the extent of activation of CaMKII by β-adrenergic stimulation and the precise signaling pathways involved are still incompletely understood, it is also accepted that CaMKII is involved in the inotropic and lusitropic effects of sympathetic stimulation (Grimm and Brown, 2010). Therefore, RyR2 proteins are natural targets of PKA and CaMKII and indeed, stimulation of beating hearts with β-adrenergic agonists readily triggers phosphorylation of RyR2 (Takasago et al., 1989; Witcher et al., 1991; Benkusky et al., 2007). Which kinase phosphorylates RyR2 to a greater extent? As early as 1989 (and before the controversy surrounding the stoichiometry of CaMKII and PKA phosphorylation of RyR2 that started in 2000, see below), Takasago et al. (1989) reported that the extent of RyR2 phosphorylation by CaMKII was ~4 times greater than that of PKA in dog hearts. This was confirmed by Witcher et al. (1991) in the same animal species. Later, using phospho-specific antibodies and autoradiograms, Rodriguez et al. (2003) confirmed that CaMKII phosphorylated at least four sites per each PKA-phosphorylated site. Hence, there is significant evidence indicating that RyR2 are far better substrates for CaMKII than they are for PKA, which is not entirely surprising given that the phosphorylation consensus for CaMKII: R/K-X-X-S/T (where X is any amino acid residue) is less stringent than that for PKA: R-R-X-S/T (Pinna and Ruzzene, 1996; however, as we will see below, a CaMKII or PKA phosphorylation consensus does not necessarily result in CaMKII or PKA phosphorylation).

Despite the presence of multiple phosphorylation consensuses in each RyR2 subunit (George, 2008) and the fact that RyR2 channels are eager substrates for several kinases (see above), only three phospho-sites have been discovered to date. Let’s discuss the most salient features of each of these sites.

Serine 2808 (S2808, mouse and human nomenclature) was first discovered by Witcher et al. (1991) as a CaMKII site but later Marx et al. (2000), Wehrens et al. (2006) labeled it as an exclusive PKA site, despite the fact that RyR2 channels with specific ablation of this phospho-epitope (RyR2-S2808A) were still phosphorylated by PKA (Benkusky et al., 2007). So, a major issue with this site is whether it’s a preferred substrate for CaMKII or PKA. Since the study by Marx et al. (2000) reporting that S2808 was hyperphosphorylated in heart failure patients and that its phosphorylation enhanced dramatically the activity of RyR2 channels, several groups have studied this phospho-site in detail (Jiang et al., 2002; Rodriguez et al., 2003; Stange et al., 2003; Currie et al., 2004; Ai et al., 2005; Xiao et al., 2005; Carter et al., 2006; Kohlhaas et al., 2006; Huke and Bers, 2008; MacDonnell et al., 2008; Fischer et al., 2013), with the majority of evidence pointing to S2808 being a target for PKA, CaMKII and possibly PKG. Also, most studies find S2808 constitutively phosphorylated [basal phosphorylation ~50–75% in several animal species and humans (Jiang et al., 2002; Rodriguez et al., 2003; Carter et al., 2006; Huke and Bers, 2008)], thus raising doubts that this site may be a reliable index of abnormal PKA-phosphorylation in heart failure patients. Indeed, Benkusky et al. (2007) found that mice with genetic ablation of the S2808 phospho-epitope (RyR2-S2808A) do not alter their β-adrenergic response, have cardiomyocyte function almost unchanged, and are not significantly protected against the maladaptive cardiac remodeling induced by chronic stress. Further, although PKA phosphorylation of RyR2 modified single-channel activity, its effect was modest and occurred at activating (systolic) [Ca^2+^], only, not at the expected low (diastolic) [Ca^2+^], where it would cause significant Ca^2+^ leak (Bers et al., 2003). The lack of protection against heart failure dysfunction as well as the normal β-adrenergic response of the RyR2-S2808A mice were confirmed by MacDonnell et al. (2008), Zhang et al. (2012) using the same transgenic mouse line. In the scheme of Marx et al. (2000), which is continuously validated and extended by additional reports by the same group (for example, Wehrens et al., 2006; Shan et al., 2010a, b), PKA phosphorylation of S2808 led to dissociation of FKBP12.6, which in turn destabilized the closed state of the channel and induced multiple subconductance states, overall increasing RyR2 Ca^2+^ fluxes, especially at diastolic [Ca^2+^]. However, most of the central tenets of this scheme have not been confirmed by others (reviewed by George, 2008; Currie, 2009; Eschenhagen, 2010; Bers, 2012; Valdivia, 2012). Specifically, Stange et al. (2003) found no effect of ablating the S2808 phospho-site (RyR2-S2808A) or simulating constitutive activation (RyR2-S2808D) in the activity of the channel or its affinity for FKBP12.6. That phosphorylation of RyR2 does not appear to dissociate FKBP12.6 was also found by Xiao et al. (2004), Guo et al. (2010). Also, there are no reports, except by the Marks’ group, indicating that RyR2 phosphorylation induces subconductance states, presumably the hallmark of FKBP12.6 dissociation. Lastly, several groups have independently reported that FKBP12.6 does not affect RyR2 activity at all (Timerman et al., 1996; Xiao et al., 2007) or that it has modest effects, only (Guo et al., 2010). Thus, although it is indisputable that S2808 is phosphorylated by PKA, CaMKII and possibly other kinases, the functional output of such reaction has been difficult to pin down and is the subject of intense debate. Further studies are needed and should continue to provide insights as drugs designed to prevent FKBP12.6 dissociation enter clinical trials and testing in humans.

Serine 2814 (S2814) was discovered by Wehrens et al. (2004) as a CaMKII site and, although there is consensus that CaMKII is the preferential kinase for this site, there is less agreement that it is *the only* CaMKII site in RyR2. As mentioned above, S2808 is also a target for CaMKII, and since there are at least ~4 CaMKII sites for each PKA site, this suggests that there are still other CaMKII sites yet to be discovered. Although S2814 is only six amino acid residues downstream of S2808 and forms part of the same RyR2 “phosphorylation hot spot” (Valdivia, 2012; Yuchi et al., 2012) the role of S2814 has been less controversial because so far it appears to be a more specific substrate for CaMKII, despite the fact that it forms part of a non-canonical CaMKII phosphorylation consensus: ^2805^RRISQTSQVSV^2815^ (S2808 and S2814 are underlined). Interestingly, another serine residue within that hot spot, S2811, is also part of a non-canonical CaMKII consensus and yet, it appears to be phosphorylated *in vitro* by PKA and CaMKII (Yuchi et al., 2012) and *in vivo* in mice stimulated by β-adrenergic agonists (Huttlin et al., 2010). Whether S2811 contributes to the effect of CaMKII phosphorylation of RyR2, or distorts the signal of phospho-specific antibodies pS2808 and pS2814 (Huke and Bers, 2008), making it difficult to discern the specificity of kinases for these phospho-epitopes, is still unclear. In quiescent cardiomyocytes, S2814 is barely phosphorylated (unlike S2808), and although its activity-dependent phosphorylation may be prevented by CaMKII inhibitors, the basal phosphorylation at rest is maintained by a Ca^2+^-dependent kinase other than CaMKII (Huke and Bers, 2008). Thus, at least two Ca^2+^-dependent kinases phosphorylate S2814, possibly leading to the same functional output (discussed below). Whether S2814 phosphorylation contributes to the inotropic effects induced by β-adrenergic stimulation has not been firmly established. However, mice with germline ablation of the S2814 phospho-epitope (RyR2-S2814A) appear more resilient than WT against a variety of cardiac insults. van Oort et al. (2010) found that RyR2-S2814A mice were protected from catecholaminergic- and pacing-induced tachyarrhythmias, whereas Ather et al. (2013) reported that arrhythmogenic spontaneous Ca^2+^ waves that were prevalent in the *mdx* mice (Duchenne muscular dystrophy model), were suppressed by crossbreeding with the RyR2-S2814A mice. In a protocol of ischemia-reperfusion injury, the RyR2-S2814A mice exhibited significantly fewer premature beats (that could be ascribed to delayed afterdepolarizations) than WT, a protection that was not seen in mice with ablation of two phospholamban phospho-sites (PLB-DM; Said et al., 2011). Respress et al. (2012) reported by RyR2-S2814 mice fared much better than WT after TAC (transverse aortic constriction)-induced heart failure, but interestingly, were not protected against MI (myocardial infarction)-induced heart failure, proposing that CaMKII phosphorylation of RyR2 plays a role in non-ischemic forms of heart failure, only. Purohit et al. (2013) reported that CaMKII activation and phosphorylation of S2814 were required to induce atrial fibrillation in angiotensin-infused mice. Thus, several laboratories (albeit all of them using the same mouse line) have independently bestowed on S2814 a preponderant role in cardiac protection, wherein inhibition of S2814 phosphorylation averts the functional and structural damage to the heart induced by heart failure, atrial fibrillation, and other insults. Since it appears illogical that S2814 phosphorylation, a seemingly common reaction, was naturally designed to wreak havoc in the heart’s function and structure, it is therefore important to demarcate the limits in which S2814 phosphorylation turns pathogenic. Interestingly, mice with constitutive activation of S2814 (S2814D), have structurally and functionally normal hearts without arrhythmias (van Oort et al., 2010) which is surprising in the context of the presumably malicious role played by S2814 phosphorylation described above.

Finally, Serine 2030 (S2030) was found by Xiao et al. (2005) using classical phospho-epitope mapping. Although S2030 is squarely within a CaMKII phosphorylation consensus (^2027^R-L-L-S^2030^), oddly this site is preferentially phosphorylated by PKA, at least *in vitro*. This site, therefore, like the other sites discussed above, does not follow *in silico* predictions of kinase specificity and conforms instead to the cryptic rules of steric hindrance, topological association to specific kinases, substrate availability, etc. that operate *in vivo* and separate *predicted* from *actual* phosphorylation sites. Experiments *in vitro* revealed a major role for S2030 in the control of RyR2 activity, and in heart failure patients, it appeared as a reliable marker of RyR2 dysfunction (Xiao et al., 2006), presumably even superseding S2808 (Marx et al., 2000). The RyR2 channel appears to be completely unphosphorylated at S2030 in quiescent cardiac myocytes, and phosphorylation is promoted by β-adrenergic stimulation (Huke and Bers, 2008), in line with *in vitro* experiments that indicate PKA phosphorylation of this site. However, the precise role of S2030 in intracellular Ca^2+^ homeostasis and cardiac performance, and its involvement in pathological states of the heart are not well understood yet. Mouse lines with genetic ablation of this site (RyR2-S2030A) have just been generated (Camors et al., 2014) and should shed light on the functional role of this novel phopho-site.

### FACTORS THAT COMPLICATE INTERPRETATION OF RyR2 PHOSPHORYLATION EFFECTS

Many lines of evidence demonstrate that RyR2 channels are phosphorylated *in vitro* and *in vivo*, but a fundamental question still pervades the field: what is the functional effect of RyR2 phosphorylation? This appears as a simple question, set for a straightforward answer, but examining the diverse and apparently opposite effects that have been published in the topic in the last ~2 decades, the only safe response seems to be that phosphorylation does *something* to RyR2 activity. All potential functional outcomes for RyR2 phosphorylation (increase, decrease, and no effect on activity) have been reported, with tantalizing hints toward, but no clear factors responsible for, the radical differences. Also, the question needs to be framed in a specific integrative context for a defined response to hold some truth. For example, whereas there is compelling evidence that PKA can alter RyR2 activity *at the single channel level* (Hain et al., 1995; Valdivia et al., 1995; Marx et al., 2000; Jiang et al., 2002; Uehara et al., 2002; Carter et al., 2006; Wehrens et al., 2006; Benkusky et al., 2007; Li et al., 2013), multiple self-regulatory systems operating in intact cells (not to mention the whole heart) may bring down this response to undetectable levels so that the answer from the cellular viewpoint would appear to be “no effect.”

Although not always sure foretellers of a defined outcome, there are some factors that modify the activity of RyR2s and are likely to complicate their response to phosphorylation. First, as stated above, RyR2 channels contain multiple phosphorylation sites that, depending on their phospho-state, may attenuate or synergize the effect of the other sites, or may require prior phosphorylation to activate the whole protein. This has become evident in experiments in which it has been possible to link variable levels of phosphorylation with defined single-channel activity (Carter et al., 2006) and also where it is clear that *phosphatases* activate RyR2 channels to higher levels than either PKA or CaMKII alone (Lokuta et al., 1995; Terentyev et al., 2003), suggesting that dephosphorylation uncovers a set of phospho-sites that modulate RyR2 activity but are not affected by either kinase. Second, RyR2 activity has long been established to be dependent on the speed of Ca^2+^ application [as inferred by Fabiato in his classical experiments that characterized CICR (Fabiato, 1985) and demonstrated in single channel experiments (Gyorke and Fill, 1993)], and this in turn may greatly influence the overall effect of phosphorylation (Valdivia et al., 1995; Jiang et al., 2002). For example, PKA phosphorylation of RyR2 channels increases their transient component of activity (peak activation) and accelerates their rate of adaptation to a steady level of activity. In cellular settings, this effect would translate into faster rates of Ca^2+^ release in response to a fast and transient Ca^2+^ entry (such as *I*_Ca_) and into little effects on steady RyR2 activity. In fact, experiments in which SR Ca^2+^ load and *I*_Ca_ were kept constant showed that β-adrenergic stimulation of ventricular myocytes accelerated the rate of Ca^2+^ release (Ginsburg and Bers, 2004), and in permeabilized cells with constant [Ca^2+^], PKA had little effect on RyR2 activity (Li et al., 2002). Thus, not all effects of RyR2 phosphorylation are detectable at constant [Ca^2+^], the preferred method of testing for such effects; fast and transient Ca^2+^ stimuli are required to unveil some of its most critical effects. Third, there exist self-correcting mechanisms that preclude persistent activation (or inhibition) of RyR2s in intact cells and can mitigate effects of phosphorylation in a few beats (Eisner et al., 1998). These mechanisms invoke restoration of steady-state Ca^2+^ fluxes when a single component of the excitation–contraction coupling machinery is perturbed or malfunctions. Thus, in its simplest terms, if the effect of PKA phosphorylation of RyR2 channels was to cause a persistent Ca^2+^ leak (as postulated by Marx et al., 2000), then the persistent leak of Ca^2+^ would necessarily cause at least partial SR Ca^2+^ depletion, which in turn would re-tune Ca^2+^ release and stop the leak. Thus, again, RyR2 responses to phosphorylation that are discretely detected at the single channel level for a relatively long period of time, may be short-lived or undetectable in cellular settings due to self-correcting mechanisms. Lastly, other factors such as Mg^2+^ (Li et al., 2013) and luminal [Ca^2+^] (Xiao et al., 2005) seem to be required in just about the right quantity for phosphorylation to exert maximal effects. Overall then, the response of RyRs to phosphorylation is neither simple nor monotonous; it is complicated by factors intrinsic and extrinsic to the channel protein and depends critically on the context (molecular, cellular, whole heart) in which it is examined.

### OVERVIEW OF CURRENT MODEL OF RyR2 PHOSPHORYLATION

**FIGURE 1:**
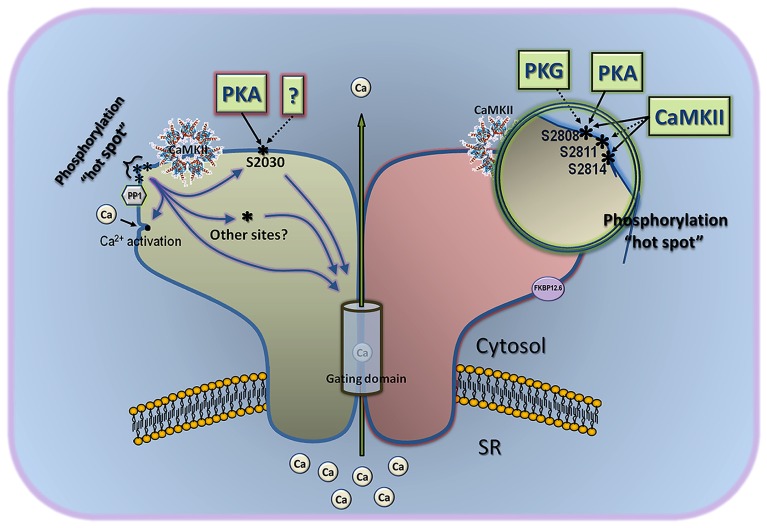
**Multi-site model of RyR2 phosphorylation.** This model considers the three phospho-sites known to date, and gives also significant weight to other as-yet-uncovered sites. The classical sites S2808 and S2814 are part of a “phosphorylation hotspot” that is located in a protruding part of the channel, is targeted by several kinases, and may contain other phospho-epitopes not yet characterized (for example, S2811). Phosphorylation of individual residues within this hotspot may be undistinguishable by the channel’s gating domain, but gradual addition of phosphate groups here may contribute to a tunable effect instead of an all-or-none response. Assuming that the phosphorylation hotspot works collectively toward a single effect, the differential regulation of PKA and CaMKII on channel gating may come about by the combined effect of each kinase on phospho-residues of the hotspot and other phosphorylation sites, such as S2030. This model also accommodates solo effects of S2030 or the phosphorylation hotspot on gating domains of the channel, as well as indirect effect on gating via interaction with classical Ca^2+^ activation sites. CaMKII and protein phosphatase 1 (PP1) are depicted close to the phosphorylation hotspot because the latter is readily phosphorylated/dephosphorylated by endogenous CaMKII and PP1. Demonstrated effect of kinases on S2808, S2814, and S2030 in intact cells or hearts is shown with a solid line, and *in vitro* effect is shown with a broken line.

Despite the recognized difficulty in linking RyR2 phosphorylation with a defined functional output, the current model of RyR2 phosphorylation is extremely simple, and needs revision. In its simplest version, PKA phosphorylation of S2808 dissociates FKBP12.6 and activates the channel by inducing long-lived subconducting states, whereas CaMKII phosphorylation of S2814 activates the channel by a different mechanism (Marx et al., 2000; Wehrens et al., 2004). However, this model disregards the S2030 phospho-site, a *bona fide* PKA site (Xiao et al., 2006), and does not give weight to the potential contribution of *other* phospho-sites, hitherto uncovered but convincingly supported by several lines of evidence. This model also does not bode well with recent structural data, wherein S2808 and S2814, by virtue of its immediacy (only six residues apart in a ~5000 amino acid subunit), form part of a larger “phosphorylation hotspot” that encompasses S2811 and possibly T2810 (^2805^RRI**S**Q**TS**QV**S**V^2815^; Yuchi et al., 2012). This phosphorylation hotspot is harbored in a flexible loop connecting two symmetrical repeats interacting with one another through β strand interactions (Yuchi et al., 2012). Because the flexible loop is exposed to solvent and protrudes prominently on the RyR2 surface, it is not surprising that the whole phosphorylation hotspot may be easily accessed by several kinases. Because of the tight clustering of phospho-sites in this hotspot, there emerges the interesting possibility that they all provide *functional redundancy*, i.e., phosphorylation of S2808 may lead to the same downstream effects as phosphorylation of S2811, or S2814. In this scenario, the sequential addition of phosphate groups to the hotspot would lead to a graded (instead of all-or-none) response of RyR2 to kinases, which could explain the widely variable response detected by investigators to a specific phosphorylation maneuver. Of course, this alternative model based on the phosphorylation hotspot alone does not explain the distinct effect of CaMKII and PKA on RyR2 activity (see below), and to fill this void it is necessary to invoke the participation of S2030 and likely other as-yet-unrecognized phospho-sites. Thus, in an obligatorily more complex model (**Figure 1**) that we call “multi-phosphosite model” (Valdivia, 2012), the differential effect of PKA and CaMKII on RyR2 activity is dictated by the integrated response of the phosphorylation hotspot *and* of additional phosphorylation sites. For example, phosphorylation of S2808 and S2030 by PKA could coordinate channel openings in response to fast calcium stimuli (Valdivia et al., 1995; Ginsburg and Bers, 2004), and phosphorylation of S2814 and other CaMKII site(s) could open RyR2s at diastolic [Ca^2+^], which would translate in Ca^2+^ leak. Certainly, until we understand the molecular basis by which the phosphorylation hotspot and other phospho-sites talk to the channel’s gating domains, this structurally-based model will remain speculative and incomplete. Nonetheless, it takes into consideration compelling evidence on the existence of various phosphorylation sites and departs substantially from the simplified notion that one kinase phosphorylates one site and produces one effect.

### CaMKII PHOSPHORYLATION OF ISOLATED RyR2 CHANNELS

Ryanodine receptor 2 channels have long been known to be a suitable substrate for CaMKII. In fact, in 1984, long before the controversies surrounding RyR2 phosphorylation and before the purification of RyR2, Seiler et al. (1984) observed that “high molecular weight proteins,” later identified as RyR2 channels, were excellent substrates for CaMKII. In the experiments of Marx et al. (2000), where RyR2 was initially presented as a structural scaffold for multiple accessory proteins, CaMKII was *not* identified as part of that macromolecular complex. However, Currie et al. (2004) and J.H. Brown’s group (reviewed in Grimm and Brown, 2010), have provided ample evidence that CaMKII is intimately associated to RyR2 (although the actual biding site is not known), logically portending an important effect of CaMKII on modulation of RyR2 activity. At the single channel level, the preponderance of results suggests that CaMKII *activates* RyR2 channels, but again, the results are not unanimous. Let’s briefly review the reports.

Although indirect evidence of RyR2 modulation by CaMKII was initially obtained by [^3^H]ryanodine binding assays (Takasago et al., 1989), the first direct demonstration was provided by Witcher et al. (1991) using single channel recordings of canine RyR2 channels reconstituted in lipid bilayers. These authors observed *activation* of RyR2 channels by both, endogenous (cardiac SR-resident) and exogenous (purified from brain homogenates) CaMKII, and correlated their results with biochemical assays showing additive levels of phosphorylation by endogenous and exogenous CaMKII. This was also the report that unveiled S2808 (S2809 in dogs) as a major CaMKII site, although the proportion of the total RyR2 phosphorylation for which S2808 was solely responsible could not be determined. Thus, if the endogenous CaMKII of Witcher et al. (1991) is the same CaMKII intimately attached to RyR2 (Currie et al., 2004; Grimm and Brown, 2010), it appears that this CaMKII phosphorylates a subset of RyR2 phospho-sites only, fewer than what is actually possible by exogenous CaMKII. Thus, the recurring point that emerges is that multiple CaMKII sites concur in the RyR2 protein, each potentially exerting a defined level of control in the channel and not always phosphorylated by a given experimental condition. This might have been why Lokuta et al. (1995) detected *inhibition* of single RyR2 channel activity by exogenous CaMKII, similar to Takasago et al. (1989), who used exogenous CaMKII in [^3^H]ryanodine binding experiments. Nevertheless, multiple CaMKII sites as justification for apparent discrepancies may not be universally applied, as Hain et al. (1995) found activation of RyR2 channels by CaMKII, using methods and animal species (dog) similar to Lokuta et al. (1995). The four studies mentioned above [Witcher et al. (1991), Lokuta et al. (1995), Takasago et al. (1989), Hain et al. (1995)] all used high (μM) [Ca^2+^] to keep the RyR2 channels open, and none of them frontally addressed the question of whether CaMKII activates RyR2 at low (diastolic) [Ca^2+^], a question of paramount importance given the current thinking that CaMKII increases SR Ca^2+^ leak (see below). This was technically difficult at the time because CaMKII itself was known to require Ca^2+^ as a cofactor for activation, and the Ca^2+^-free, auto-phosphorylated active form of the enzyme was not widely known. Hence, more studies are needed to clarify the Ca^2+^-dependence of CaMKII effect on isolated RyR2 channels. Lastly, in an oversimplified scheme that largely ignored the overwhelming evidence for the aforementioned multiple CaMKII sites, Wehrens et al. (2004) found that CaMKII activated single RyR2 channels and postulated that ablation of a single phospho-site (S2815) was sufficient to inhibit CaMKII phosphorylation completely. In summary, CaMKII phosphorylation of isolated RyR2 channels is readily detected at the biochemical level, and the preponderance of results indicate that CaMKII activates RyR2 channels by targeting multiple sites; however, some studies find that CaMKII may inhibit RyR2 channel activity and the nature of this apparent discrepancy is not easily explained. Furthermore, whether CaMKII phosphorylation activates individual RyR2 channels at low (diastolic) [Ca^2+^] has not been firmly established yet and needs more refined studies.

### EFFECT OF CaMKII ON SR Ca^2+^ RELEASE

Many studies have addressed the role of CaMKII phosphorylation in SR Ca^2+^ release and excitation–contraction coupling of intact ventricular myocytes, and most of them are detailed in excellent reviews (George, 2008; Currie, 2009; Grimm and Brown, 2010; Currie et al., 2011; Luo and Anderson, 2013; Bers, 2014). Here we will simplify the discussion by concentrating on the studies that have addressed the role of CaMKII phosphorylation on the most direct indicators of RyR2 function, namely, SR Ca^2+^ leak, spontaneous Ca^2+^ waves, and Ca^2+^ sparks. This circumvents the problem of interpreting CaMKII effects on RyR2 based on whole cell results or global Ca^2+^ transients, which are the product of multiple nodes of activity interacting in complex ways.

Most studies find that β-adrenergic stimulation increases SR Ca^2+^ leak, and that chronic adrenergic stimulation of ventricular myocytes such as that occurring in heart failure produces a cellular substrate favorable for generation of Ca^2+^-triggered arrhythmias. To what extent are CaMKII and RyR2 channels responsible for these effects? We examined in preceding paragraphs that, although the precise transduction pathways have not been completely elucidated yet, it is clear that CaMKII is activated upon β-adrenergic stimulation of the heart (Grimm and Brown, 2010). In addition, a significant number of studies [but not all (Yang et al., 2007)] find that CaMKII activation increases SR Ca^2+^ leak (Maier et al., 2003; Currie et al., 2004; Guo et al., 2006; Curran et al., 2007) and that the SR Ca^2+^ leak that is characteristically increased in heart failure (Kirchhefer et al., 1999; Marx et al., 2000; Ai et al., 2005) may be prevented by specific CaMKII inhibition (Wu et al., 2002; Ai et al., 2005; Curran et al., 2010; Sossalla et al., 2010; Respress et al., 2012), but not by PKA inhibition (Curran et al., 2010). Hence, making the reasonable assumption that RyR2 channels are the main pathway for SR Ca^2+^ leak, and deriving from the reports above that CaMKII activation evokes arrhythmogenic SR Ca^2+^ leak, then it is fair to conclude that CaMKII phosphorylation *activates* RyR2 at diastolic [Ca^2+^] to produce unchecked SR Ca^2+^ release that is capable of bringing membrane potential to threshold (delayed afterdepolarizations) and quite possibly ignite cellular pathways that lead to cardiac remodeling. From this perspective, CaMKII is presented as an arrhythmogenic, deleterious kinase, and RyR2 its main instrument of deraignment. Obviously, the seemingly belittled positive effect of CaMKII in normal cell function cannot be discounted, and a balance between physiological and pathological effects of CaMKII activation must exist *in vivo*. Thus, an emerging notion is that normally, *acute* CaMKII and PKA activation result in an increased magnitude and rate of Ca^2+^ release, respectively (Ginsburg and Bers, 2004), which account in no small part for the inotropic effects of β-adrenergic stimulation. This hypothesis is supported by studies that find that CaMKII increases fractional Ca^2+^ release (reviewed in Anderson et al., 2011) and that PKA increases the rate of Ca^2+^ release, only, in cells with controlled L-type Ca^2+^ channel trigger and SR Ca^2+^ content (Ginsburg and Bers, 2004). The role of CaMKII in force-frequency stimulation is also well known (Krishna et al., 2013). On the other hand, it appears that most of the deleterious effects of CaMKII are exerted under conditions that allow its *chronic* activation. It has become evident that CaMKII is not only a sensor of Ca^2+^ signals, but it is also exquisitely sensitive to oxidative stress (Luczak and Anderson, 2014). Oxidative stress is an important ingredient of the pathogenic recipe that deranges cardiomyocytes in atrial fibrillation, heart failure, sinus node dysfunction, and other cardiomyopathies. Thus, persistent activation of CaMKII by reactive oxygen species (ROS), is an expected (and demonstrated) side effect of many cardiac insults, and a constitutively activated CaMKII has an ample range of action (hence the name multi-functional), including several ion channels and transporters that control membrane excitability and excitation–contraction coupling (Bers and Grandi, 2009), and others that control excitation–transcription coupling (Bers, 2011). Since, as we noted above, RyR2 channels are natural and avid substrates of CaMKII and are themselves affected by oxidative stress (Donoso et al., 2011), the contribution of RyR2 to altered Ca^2+^ homeostasis in these cardiac pathologies is almost assured. Overall, then, CaMKII walks a fine line separating “good” from “evil,” and a great part of this dichotomy is dictated by its effect on RyR2 channels and its capacity to induce (potentially excessive) SR Ca^2+^ leak. Studies aimed at demarcating the pivotal point in which CaMKII contributes to health or disease continues at great strides. Until then, inhibiting CaMKII phosphorylation of RyR2 channels as targeted approach to prevent the excessive Ca^2+^ leak and the spontaneous Ca^2+^ waves that undergird several cardiomyopathies appears enticing, but needs further studies.

## CaMKII REGULATION OF INOSITOL 1,4,5-TRIPHOSPHATE RECEPTORS

### GENERAL CONSIDERATIONS

In the majority of mammalian cells, complex intracellular Ca^2+^ signals elicited by neurohormonal stimuli are mediated through the generation of inositol 1,4,5-triphosphate (InsP_3_) and the activation of its receptor, the InsP_3_R. InsP_3_ originates from the hydrolysis of the membrane phospholipid phosphatidylinositol 4,5-bisphosphate (PIP2) by phospholipase C (PLC; reviewed in Foskett et al., 2007; Taylor and Tovey, 2010). Concomitant binding of InsP_3_ and Ca^2+^ is necessary for InsP_3_R activation, and this in turn leads to Ca^2+^ release within the cytoplasm. Multiple stimuli by a given agonist result in repetitive InsP_3_R-mediated Ca^2+^ releases (Ca^2+^ oscillations) that propagate as Ca^2+^ waves through the entire cell first and may ultimately stimulate neighboring cells to form inter-cellular Ca^2+^ waves. In cardiac myocytes, localization of InsP_3_Rs in the nuclear envelop underlies their role in excitation–transcription coupling, but recent evidence (mostly from atrial cells) suggest that although their abundance is low in the t-tubules and the sarcolemma, InsP_3_Rs can also rev up excitation–contraction coupling and eventually trigger cellular arrhythmias (Mackenzie et al., 2002; Zima and Blatter, 2004; Li et al., 2005). Similar to RyR2 channels, InsP_3_Rs are scaffolding proteins highly regulated by ions (Ca^2+^, H^+^), nucleotides (ATP), accessory proteins (FKBP12, calmodulin), and also undergo major post-translational modifications such as phosphorylation (Taylor and Tovey, 2010). The first evidence for InsP_3_Rs phosphorylation was obtained in the late 80s by Greengard’s and Sydner’s groups from rat cerebella (Supattapone et al., 1988; Walaas et al., 1988). Today, at least 15 different kinases and phosphatases, including CaMKII, are postulated to target the InsP_3_Rs and regulate their activity in an isoform- and tissue-specific manner. This second part of this review will focus on the newly discovered CaMKII site (Ser150), on the physiological consequences of CaMKII phosphorylation of InsP_3_R, and on the mechanisms by which this event may participate in cardiac Ca^2+^ signaling. Further details on the general properties of InsP_3_R and their role in cardiac myocytes may be found in excellent reviews (Foskett et al., 2007; Kockskämper et al., 2008; Vanderheyden et al., 2009; Taylor and Tovey, 2010).

### InsP_3_R CHANNEL: FROM STRUCTURE TO FUNCTION

In mammals, three different genes (*ITPR1*, *ITPR2,* and *IPTR3*) encode for ~300 kDa subunits that assemble as homo- or hetero-tetramers to form a functional InsP_3_R channel. Similar to RyR2, the low resolution of InsP_3_Rs crystals shows a mushroom-like structure with a large cytosolic *cap* constituted by the N-terminal and central regions of the protein and a short *stem*, inserted into the membrane by the C-terminal domain (Da Fonseca et al., 2003). The InsP_3_-binding domain (or “core”) is located within the first amino acids of the channel, while up to eight Ca^2+^ binding sites are distributed throughout its entire sequence (including two sites within the InsP_3_-binding core; Mignery et al., 1990; Mignery and Sudhof, 1990; Pietri et al., 1990; Sienaert et al., 1996, 1997). The Ca^2+^ pore is formed by a classic P-loop between the membrane-spanning segments 5 and 6. Altogether the organization of InsP_3_Rs suggests that the opening of the channel depends on large allosteric movements that, by analogy with the RyR2s, may be finely tuned by phosphorylation (Van Petegem, 2012).

Depending on the subtype, the apparent *Kd* of the receptors for InsP_3_ varies from 10 to 80 nM (with InsP_3_R-2>>InsP_3_R-1>InsP_3_R-3; Newton et al., 1994). Remarkably, the binding of InsP_3_ to the receptor channel is stoichiometric and regulates the properties of the InsP_3_R-mediated Ca^2+^ release. At low intracellular concentrations, InsP_3_-binding induces the opening of single InsP_3_R channel promoting a unitary Ca^2+^ release (dubbed “Ca^2+^ blip”). Increasing InsP_3_ concentrations facilitate the opening of clusters of InsP_3_Rs, allowing CICR within a cluster. At high concentration, InsP_3_ promotes CICR between the clusters and intracellular Ca^2+^ waves (Bootman et al., 1997). A noteworthy property of the InsP_3_Rs is their high affinity for Ca^2+^, causing maximal activity at diastolic [Ca^2+^]_i_ (100 nM) while in similar conditions RyR2 are closed (Ramos-Franco et al., 1998). Therefore in myocytes stimulated by Gq–protein coupled receptor (GqPCR) agonists, InsP_3_Rs-mediated Ca^2+^ release may occur during diastole and “prime” the RyR2 to open, thus potentially increasing RyR2-mediated SR Ca^2+^ leak and triggering of arrhythmias (Mackenzie et al., 2002; Zima and Blatter, 2004; Li et al., 2005).

### EFFECT OF CaMKII PHOSPHORYLATION ON InsP_3_R ACTIVITY

Since the first reports of InsP_3_R phosphorylation more than two decades ago (Supattapone et al., 1988; Walaas et al., 1988), the growing number of kinases that target InsP_3_Rs and either decrease or increase their activity have uncovered the complexity of InsP_3_Rs regulation. Among those enzymes, recent observations demonstrate that CaMKII plays a critical role for InsP_3_R function.

**FIGURE 2:**
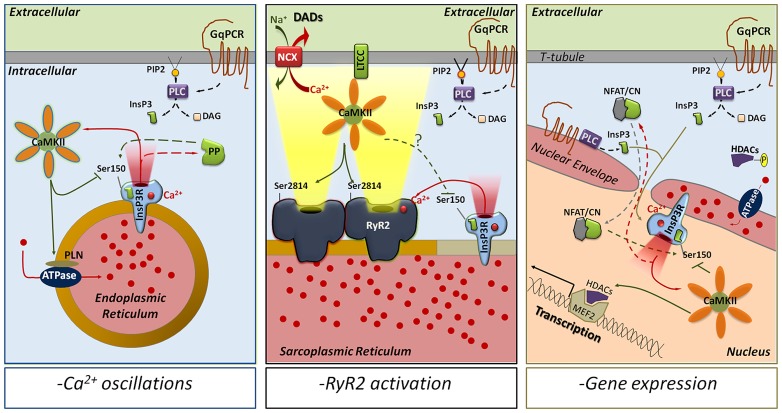
**CaMKII phosphorylation of Ser150 as a modulator of InsP_3_R-mediated Ca^2+^ release.** The activation of GqPCRs leads to the production of InsP_3_ by PLC and initiates InsP_3_R-mediated Ca^2+^ release. The released Ca^2+^ activates CaMKII, which in turn phosphorylates InsP_3_R-S150 to inhibit channel function. Depending on the intracellular location, CaMKII phosphorylation of InsP_3_Rs could be implicated in multiple responses. In the endoplasmic reticulum of most of the mammalian cells (*left panel*), CaMKII phosphorylation of S150 alternates with protein phosphatases dephosphorylation to trigger intracellular Ca^2+^ oscillations and mediate complex Ca^2+^ signals. In the SR of cardiac myocytes (*middle panel*), InsP_3_Rs are able to activate RyR2s by CICR even though they seem to be excluded from the dyadic cleft. This increases Ca^2+^ transients and might trigger delayed afterdepolarizations (via NCX activation). CaMKII phosphorylation of InsP_3_R-S150, which has not been determined yet, could then prevent the uncontrolled activation of RyR2s and decrease the incidence of arrhythmias. In the nucleus (*right panel*), InsP_3_R Ca^2+^ release activates CaMKII, which phosphorylates HDAC proteins to promote transcription. S150- phosphorylation by CaMKII could limit InsP_3_Rs activation in time and space. However, if associated with calcineurin-dephosphorylation of S150, CaMKII could favor InsP_3_R-dependent Ca^2+^ oscillations as secondary mechanism to activate gene expression. ATPase, sarco/endoplasmic reticulum Ca^2+^ ATPase; NFAT/CN, NFAT-calcineurin complex; DAD, delayed after depolarization; DAG, diacylglycerol; GqPCR, Gq–protein coupled receptor; HDAC, histone deacetylase; LTCC, L-type Ca^2+^ channel; MEF2, myocyte enhancer factor-2; NCX, Na^+^/Ca^2+^ exchanger; PLC, phospholipase C; PIP2, phosphatidylinositol 4,5-bisphosphate; PP, protein phosphatase (1, 2A, and/or 2B).

The consequences of CaMKII phosphorylation of InsP_3_R were first ascertained in permeabilized embryonic mouse fibroblasts (Zhang et al., 1993). In these cells, CaMKII phosphorylation permits the activation of the InsP_3_R channel upon addition of InsP_3_ or an agonist cocktail (bradykinin + GTPγS). This activation takes place at intracellular [Ca^2+^] ranging from 30 to 100 nM. At higher [Ca^2+^]_i_, calcineurin activity prevails and dephosphorylation of the channel occurs. In agreement with these results, Cameron et al. (1995) observed that CaMKII enhanced the InsP_3_-sensitive Ca^2+^ flux in rat cerebellum microsomes. However, these outcomes were challenged by observations made in intact HeLa cells stimulated by histamine (Zhu et al., 1996). In this model, it is the Ca^2+^ released by the InsP_3_R that activates CaMKII, which phosphorylates back InsP_3_R to terminate the release. In a second step, the activation of a phosphatase (likely PP1/PP2A) dephosphorylates InsP_3_Rs to restore its activity. This model is particularly elegant since the authors also demonstrated that activated CaMKII facilitates the ER Ca^2+^ refilling by increasing SERCA activity (likely through PLN phosphorylation). As a consequence, the alternation between phosphorylated and dephosphorylated states of InsP_3_Rs seemed to be the basic mechanism for histamine-induced intracellular Ca^2+^ oscillations (**Figure 2**, *left panel*). Later, similar inhibitory effects of InsP_3_Rs phosphorylation by CaMKII were reported in channels reconstituted in lipid bilayers (Bare et al., 2005) as well as in *Xenopus* oocytes (Matifat et al., 2001) and bovine endothelial cells (Aromolaran and Blatter, 2005). Today, the origin of these conflicting observations remains unclear but a fair approximation is that it is linked to the high variability of the InsP_3_Rs sequence induced by alternative splicing, or to tissue-specific expression of accessory proteins (Foskett et al., 2007).

Recently, Mignery et al. (1990), Maxwell et al. (2012) identified Ser150 (S150) as a distinct CaMKII phosphorylation site on InsP_3_R-2s. They demonstrated in lipid bilayers that the replacement of S150 by the non-phosphorylatable residue alanine (InsP_3_R-2^S150A^) abrogates the inhibitory effects of CaMKII phosphorylation. Conversely, the phospho-mimetic mutant InsP_3_R-2^S150E^ reproduced the blunted channel activity of phosphorylated WT receptors. Noticeably, S150 belongs to a suppressor domain located prior to the InsP_3_-binding core. The ablation of that domain increases InsP_3_R affinity for InsP_3_ but additionally blocks Ca^2+^ release by the channel (Uchida et al., 2003). The proposed mechanism is that the suppressor domain participates in the transduction of the allosteric movements that are necessary for channel opening (Uchida et al., 2003). Therefore, it is possible that phosphorylation of S150 by CaMKII also blocks the transmission of the activation signal to the pore domain rather than decreasing the affinity of the InsP_3_Rs for the InsP_3_. This hypothesis is reinforced by the fact that high [InsP_3_]_i_ is not able to reverse the inhibition of InsP_3_Rs activity after CaMKII phosphorylation (Zhu et al., 1996).

### InsP_3_R IN THE HEART: CONSEQUENCES OF CaMKII PHOSPHORYLATION

The mammalian heart expresses all three InsP_3_R isoforms. InsP_3_R-1 is abundant in endothelial cells and Purkinje fibers. InsP_3_R-2 is the main isoform of cardiac myocytes and pacemaker cells. Finally, InsP_3_R-3s are present in all cell types but at a lower level (5–15% total InsP_3_R expression; Perez et al., 1997; Lipp et al., 2000; Ju et al., 2011). Although the roles of InsP_3_Rs in the cardiac function have been overlooked, recent evidence assigns to InsP_3_R-2s an important role in pathologic hypertrophy and Ca^2+^-triggered arrhythmias.

#### InsP_3_R-2, CaMKII, and excitation–transcription coupling

In both atrial and ventricular myocytes, InsP_3_R-2s are mainly present in the nuclear envelope where their implication in pathological excitation–transcription coupling has been recently uncovered (reviewed in Kockskämper et al., 2008). During cardiac hypertrophy induced by pressure overload or chronic activation of the GqPCRs, the InsP_3_R-2s activation leads to a subsequent release of Ca^2+^ in the nucleoplasm, which activates the nuclear isoform of CaMKIIδ (CaMKIIδ_B_). CaMKIIδ_B_ phosphorylates the histone deacetylases (HDACs) to induce their nuclear export and relieve their inhibition on the transcription factor MEF-2 (myocyte enhancer factor-2; Zhu et al., 2000; Wu et al., 2006; Ago et al., 2010). In this context the inhibition of the InsP_3_Rs by a CaMKIIδ_B_ feedback phosphorylation would limit in time and space the InsP_3_R-mediated Ca^2+^ release and consequently be anti-hypertrophic. Conversely, a possible pro-hypertrophic role for CaMKIIδ_B_ phosphorylation of InsP_3_R is borne out from recent observations. First, InsP_3_R-mediated Ca^2+^ release is able to activate two targets of calmodulin: CaMKIIδ_B_ and calcineurin, a perinuclear phosphatase that translocates to the nucleus complexed with the transcription factor NFAT (reviewed in Molkentin, 2000; Bootman et al., 2009). The inhibition of either CaMKII or calcineurin prevents the InsP_3_R-mediated cardiac hypertrophy (Molkentin et al., 1998; Taigen et al., 2000; Zhu et al., 2000). Second, in addition to their classical localization in the sarcolemma, GqPCRs have been recently identified in the nuclear envelope as well as in t-tubules that spread near the nucleus of cardiomyocyte (Tadevosyan et al., 2012; Ibarra et al., 2013). This allows for a rapid and local production of InsP_3_ that diffuses and signals into the nucleus independently from the cytosolic InsP_3_ concentration (Ibarra et al., 2013). Finally, Luo et al. (2008) showed that neonatal rat cardiac myocytes stimulated by a GqPCR agonist exhibit repetitive InsP_3_R-mediated Ca^2+^ waves (or Ca^2+^ oscillations) that take place in the nucleus autonomously from the cytosolic [Ca^2+^]. Interestingly, frequency-dependent Ca^2+^ oscillations have been shown to increase gene expression via the activation of the calcineurin/NF-AT pathway (Dolmetsch et al., 1998). Altogether, these observations suggest that hypertrophic signals may be generated by intra-nuclear Ca^2+^ oscillations independently (frequency, amplitude, and duration) from the excitation–contraction coupling. InsP_3_-dependent oscillations would be created through InsP_3_Rs activation–inhibition cycles mediated by calcineurin dephosphorylation and CaMKIIδ_B_ phosphorylation of S150, respectively (**Figure 2**, *right panel*).

#### InsP_3_R-2, CaMKII, and excitation–contraction coupling

In cardiac myocytes, the expression of InsP_3_R-2s is remarkably low compared to RyR2s (for an InsP_3_R-2:RyR2 ratio of 1:50 to 1:100 in ventricular myocytes). In addition, most of the immunofluorescence studies that have observed partial localization of InsP_3_R-2s in the sub-sarcolemmal space of atrial myocytes, failed to detect InsP_3_R-2s in the surface sarcolemma or t-tubules of ventricular myocytes (Bare et al., 2005; Escobar et al., 2011; except Mohler et al., 2003). Activation of InsP_3_R-2s by a GqPCR agonist produces, in the myocyte, a small and slow cytosolic Ca^2+^ spark-like release dubbed “Ca^2+^ puffs” (Kockskämper et al., 2008). Ca^2+^ puffs can activate the neighboring RyR2s to increase the Ca^2+^ spark frequency and the Ca^2+^ transient amplitude. In pathological conditions, InsP_3_R-2 mediated Ca^2+^ release can ultimately cause, in atrial cells, Ca^2+^ alternans and increased susceptibility to arrhythmias (Mackenzie et al., 2002; Zima and Blatter, 2004; Li et al., 2005). Moreover, although their expression is not detected in t-tubules, some reports support a similar role for InsP_3_R-mediated release in ventricular myocyte (Domeier et al., 2008; Signore et al., 2013 but not Mackenzie et al., 2002; Zima and Blatter, 2004; Li et al., 2005). All together, these data suggest that InsP_3_R-2s are not directly involved in excitation–contraction coupling but participate as modulators that “prime” the RyR2s to increase their sensitivity to diastolic [Ca^2+^] and LTCC current. In that context, the inhibition of InsP_3_R-dependent Ca^2+^ release following CaMKIIδ_C_ phosphorylation of InsP_3_R-Ser150 would block RyR2 potentiation and exert an anti-arrhythmic effect. However, in an extensive phospho-proteome analysis that identified the vast majority of proteins phosphorylated during β-adrenergic stimulation in mice, Huttlin et al. (2010) failed to detect phosphorylation of the cardiac InsP_3_R-2s, including S150, while InsP_3_R-1 and -3 were targeted by PKA at Ser1588 and Ser934, respectively. This suggests that the InsP_3_R-2 channels expressed in the SR are not located in the vicinity of the CaMKIIδ_C_/PKA microdomains, and therefore they may be excluded from the dyad containing the RyR2s (**Figure 2**, *central panel*). In agreement with that result, Signore et al. (2013) described in mouse ventricular myocytes that the InsP_3_R-2 effects are mediated through the activation of the NCX and the increase of the action potential duration rather than a direct effect on RyR2s. Overall, these observations suggest an absence of a direct cross-talk between RyR2s and InsP_3_Rs and advocate the hypothesis that InsP_3_Rs mediate their effects on RyR2s by limited Ca^2+^ diffusion from the InsP_3_R release sites to the RyR2 sites.

Interestingly, Huttlin et al. (2010) also identified two cardiac-specific epitopes on InsP_3_R-3 (Ser930 and Ser2189) for which the targeting kinase(s) remain(s) undetermined. Noticeably, S2189 of InsP_3_R-3 contains the CaMKII consensus sequence (Pinna and Ruzzene, 1996) and is absent from InsP_3_R-1 or -2. More studies will be necessary to determine whether this InsP_3_R-3 phosphorylation occurs in cardiac myocytes and the mechanisms by which it affects cardiac function.

## CONCLUDING REMARKS

CaMKII is a pleiotropic kinase that targets several ion channels and transporters in the heart, and RyR2 and InsP_3_R channels are among its principal substrates. Interestingly, despite the relatively strong structural similarity between these two intracellular Ca^2+^ release channels, the majority (but not all) of the studies indicate that CaMKII phosphorylation of RyR2s and InsP_3_Rs leads to antithetical outcomes. On one hand, CaMKII phosphorylation is presumed to *increase* RyR2 activity and promote SR Ca^2+^ leak that, when excessive, may trigger cardiac arrhythmias. On the other hand, CaMKII phosphorylation of nuclear InsP_3_Rs is presumed to *inhibit* InsP_3_Rs-mediated Ca^2+^ release and to prevent intra-nuclear Ca^2+^ oscillations (although cytosolic InsP_3_R-mediated Ca^2+^ release seems to promote arrhythmias, too). The magnitude of the effect of phosphorylation on these channels is also purportedly different since CaMKII *modulates* RyR2 channel activity, only, while it appears to play an on/off function on InsP_3_Rs. The bases of both of these differences are not immediately apparent and in fact, there is no universal agreement that such differences exist. As can be derived from the preceding paragraphs, a unified scheme on the effect of phosphorylation on both, RyR2 and InsP_3_Rs is yet to be forged. Nonetheless, independent of its precise mechanism of action on these channels, an emerging notion is that *excessive* CaMKII activity is detrimental for cardiac performance, and the potential salutary effect of blocking its chronic effects appears worth pursuing since its action on RyR2s and InsP_3_Rs might prevent both Ca^2+^-mediated arrhythmias and Ca^2+^-dependent activation of hypertrophic gene programs.

## Conflict of Interest Statement

The authors declare that the research was conducted in the absence of any commercial or financial relationships that could be construed as a potential conflict of interest.

## References

[B1] Ago T. Yang Y. Zhai P. Sadoshima J. (2010). Nifedipine inhibits cardiac hypertrophy and left ventricular dysfunction in response to pressure overload. *J. Cardiovasc. Transl. Res.* 3 304–313 10.1007/s12265-010-9182-x 20559781 PMC3036765

[B2] Ai X. Curran J. W. Shannon T. R. Bers D. M. Pogwizd S. M. (2005). Ca^2+^/calmodulin-dependent protein kinase modulates cardiac ryanodine receptor phosphorylation and sarcoplasmic reticulum Ca^2+^ leak in heart failure. *Circ. Res.* 97 1314–1322 10.1161/01.RES.0000194329.41863.89 16269653

[B3] Anderson M. E. Brown J. H. Bers D. M. (2011). CaMKII in myocardial hypertrophy and heart failure. *J. Mol. Cell. Cardiol.* 51 468–473 10.1016/j.yjmcc.2011.01.012 21276796 PMC3158288

[B4] Aromolaran A. A. Blatter L. A. (2005). Modulation of intracellular Ca^2+^ release and capacitative Ca^2+^ entry by CaMKII inhibitors in bovine vascular endothelial cells. *Am. J. Physiol. Cell Physiol.* 289 C1426–C1436 10.1152/ajpcell.00262.2005 16093279

[B5] Ather S. Wang W. Wang Q. Li N. Anderson M. E. Wehrens X. H. (2013). Inhibition of CaMKII phosphorylation of RyR2 prevents inducible ventricular arrhythmias in mice with Duchenne muscular dystrophy. *Heart Rhythm* 10 592–599 10.1016/j.hrthm.2012.12.016 23246599 PMC3605194

[B6] Balshaw D. M. Yamaguchi N. Meissner G. (2002). Modulation of intracellular calcium-release channels by calmodulin. *J. Membr. Biol.* 185 1–8 10.1007/s00232-001-0111-4 11891559

[B7] Bare D. J. Kettlun C. S. Liang M. Bers D. M. Mignery G. A. (2005). Cardiac type 2 inositol 1,4,5-trisphosphate receptor: interaction and modulation by calcium/calmodulin-dependent protein kinase II. *J. Biol. Chem.* 280 15912–15920 10.1074/jbc.M414212200 15710625

[B8] Benkusky N. A. Weber C. S. Scherman J. A. Farrell E. F. Hacker T. A. John M. C. (2007). Intact beta-adrenergic response and unmodified progression toward heart failure in mice with genetic ablation of a major protein kinase A phosphorylation site in the cardiac ryanodine receptor. *Circ. Res.* 101 819–829 10.1161/CIRCRESAHA.107.153007 17717301

[B9] Bers D. M. (2004). Macromolecular complexes regulating cardiac ryanodine receptor function. *J. Mol. Cell. Cardiol.* 37 417–429 10.1016/j.yjmcc.2004.05.026 15276012

[B10] Bers D. M. (2011). Ca(2)(+)-calmodulin-dependent protein kinase II regulation of cardiac excitation-transcription coupling. *Heart Rhythm* 8 1101–1104 10.1016/j.hrthm.2011.01.030 21255680 PMC3129479

[B11] Bers D. M. (2012). Ryanodine receptor S2808 phosphorylation in heart failure: smoking gun or red herring. *Circ. Res.* 110 796–799 10.1161/CIRCRESAHA.112.265579 22427320

[B12] Bers D. M. (2014). Cardiac sarcoplasmic reticulum calcium leak: basis and roles in cardiac dysfunction. *Annu. Rev. Physiol.* 76 107–127 10.1146/annurev-physiol-020911-153308 24245942

[B13] Bers D. M. Eisner D. A. Valdivia H. H. (2003). Sarcoplasmic reticulum Ca^2+^ and heart failure: roles of diastolic leak and Ca^2+^ transport. *Circ. Res.* 93 487–490 10.1161/01.RES.0000091871.54907.6B 14500331

[B14] Bers D. M. Grandi E. (2009). Calcium/calmodulin-dependent kinase II regulation of cardiac ion channels. *J. Cardiovasc. Pharmacol.* 54 180–187 10.1097/FJC.0b013e3181a25078 19333131 PMC2784004

[B15] Bootman M. D. Berridge M. J. Lipp P. (1997). Cooking with calcium: the recipes for composing global signals from elementary events. *Cell* 91 367–373 10.1016/S0092-8674(00)80420-1 9363945

[B16] Bootman M. D. Fearnley C. Smyrnias I. Macdonald F. Roderick H. L. (2009). An update on nuclear calcium signalling. *J. Cell Sci.* 122 2337–2350 10.1242/jcs.028100 19571113

[B17] Cameron A. M. Steiner J. P. Roskams A. J. Ali S. M. Ronnett G. V. Snyder S. H. (1995). Calcineurin associated with the inositol 1,4,5-trisphosphate receptor-FKBP12 complex modulates Ca^2+^ flux. *Cell* 83 463–472 10.1016/0092-8674(95)90124-8 8521476

[B18] Camors E. Loaiza R. Alvarado F. Zhao Y. Powers P. Valdivia H. H. (2014). Preventing RyR2-S2808 and RyR2-S2814 phosphorylation does not alter the β-adrenergic response of mouse hearts. *Biophys. J.* 106:108a

[B19] Capes E. M. Loaiza R. Valdivia H. H. (2011). Ryanodine receptors. *Skelet. Muscle* 1:18 10.1186/2044-5040-1-18 PMC3156641 21798098

[B20] Carter S. Colyer J. Sitsapesan R. (2006). Maximum phosphorylation of the cardiac ryanodine receptor at serine-2809 by protein kinase a produces unique modifications to channel gating and conductance not observed at lower levels of phosphorylation. *Circ. Res.* 98 1506–1513 10.1161/01.RES.0000227506.43292.df 16709901

[B21] Curran J. Brown K. H. Santiago D. J. Pogwizd S. Bers D. M. Shannon T. R. (2010). Spontaneous Ca waves in ventricular myocytes from failing hearts depend on Ca(2+)-calmodulin-dependent protein kinase II. *J. Mol. Cell. Cardiol.* 49 25–32 10.1016/j.yjmcc.2010.03.013 20353795 PMC2883657

[B22] Curran J. Hinton M. J. Rios E. Bers D. M. Shannon T. R. (2007). Beta-adrenergic enhancement of sarcoplasmic reticulum calcium leak in cardiac myocytes is mediated by calcium/calmodulin-dependent protein kinase. *Circ. Res.* 100 391–398 10.1161/01.RES.0000258172.74570.e6 17234966

[B23] Currie S. (2009). Cardiac ryanodine receptor phosphorylation by CaM Kinase II: keeping the balance right. *Front. Biosci.* 14:5134–5156 10.2741/3591 19482609

[B24] Currie S. Elliott E. B. Smith G. L. Loughrey C. M. (2011). Two candidates at the heart of dysfunction: the ryanodine receptor and calcium/calmodulin protein kinase II as potential targets for therapeutic intervention-An in vivo perspective. *Pharmacol. Ther.* 131 204–220 10.1016/j.pharmthera.2011.02.006 21414358

[B25] Currie S. Loughrey C. M. Craig M. A. Smith G. L. (2004). Calcium/calmodulin-dependent protein kinase IIdelta associates with the ryanodine receptor complex and regulates channel function in rabbit heart. *Biochem. J.* 377 357–366 10.1042/BJ20031043 14556649 PMC1223879

[B26] Da Fonseca P. C. Morris S. A. Nerou E. P. Taylor C. W. Morris E. P. (2003). Domain organization of the type 1 inositol 1,4,5-trisphosphate receptor as revealed by single-particle analysis. *Proc. Natl. Acad. Sci. U.S.A.* 100 3936–3941 10.1073/pnas.0536251100 12651956 PMC153026

[B27] Dolmetsch R. E. Xu K. Lewis R. S. (1998). Calcium oscillations increase the efficiency and specificity of gene expression. *Nature* 392 933–936 10.1038/31960 9582075

[B28] Domeier T. L. Zima A. V. Maxwell J. T. Huke S. Mignery G. A. Blatter L. A. (2008). IP3 receptor-dependent Ca^2+^ release modulates excitation-contraction coupling in rabbit ventricular myocytes. *Am. J. Physiol. Heart Circ. Physiol.* 294 H596–H604 10.1152/ajpheart.01155.2007 18055509

[B29] Donoso P. Sanchez G. Bull R. Hidalgo C. (2011). Modulation of cardiac ryanodine receptor activity by ROS and RNS. *Front. Biosci.* 16:553–567 10.2741/3705 21196188

[B30] Eisner D. A. Trafford A. W. Diaz M. E. Overend C. L O’Neill S. C. (1998). The control of Ca release from the cardiac sarcoplasmic reticulum: regulation versus autoregulation. *Cardiovasc. Res.* 38 589–604 10.1016/S0008-6363(98)00062-5 9747428

[B31] Eschenhagen T. (2010). Is ryanodine receptor phosphorylation key to the fight or flight response and heart failure? *J. Clin. Invest.* 120 4197–4203 10.1172/JCI45251 21099119 PMC2994341

[B32] Escobar M. Cardenas C. Colavita K. Petrenko N. B. Franzini-Armstrong C. (2011). Structural evidence for perinuclear calcium microdomains in cardiac myocytes. *J. Mol. Cell. Cardiol.* 50 451–459 10.1016/j.yjmcc.2010.11.021 21147122

[B33] Fabiato A. (1985). Time and calcium dependence of activation and inactivation of calcium-induced release of calcium from the sarcoplasmic reticulum of a skinned canine cardiac Purkinje cell. *J. Gen. Physiol.* 85 247–289 10.1085/jgp.85.2.247 2580043 PMC2215800

[B34] Farrell E. F. Antaramian A. Rueda A. Gomez A. M. Valdivia H. H. (2003). Sorcin inhibits calcium release and modulates excitation-contraction coupling in the heart. *J. Biol. Chem.* 278 34660–34666 10.1074/jbc.M305931200 12824171

[B35] Fill M. Copello J. A. (2002). Ryanodine receptor calcium release channels. *Physiol. Rev.* 82 893–922 10.1152/physrev.00013.2002 12270947

[B36] Fischer T. H. Herting J. Tirilomis T. Renner A. Neef S. Toischer K. (2013). Ca^2+^/calmodulin-dependent protein kinase II and protein kinase A differentially regulate sarcoplasmic reticulum Ca^2+^ leak in human cardiac pathology. *Circulation* 128 970–981 10.1161/CIRCULATIONAHA.113.001746 23877259

[B37] Foskett J. K. White C. Cheung K. H. Mak D. O. (2007). Inositol trisphosphate receptor Ca^2+^ release channels. *Physiol. Rev.* 87 593–658 10.1152/physrev.00035.2006 17429043 PMC2901638

[B38] George C. H. (2008). Sarcoplasmic reticulum Ca^2+^ leak in heart failure: mere observation or functional relevance? *Cardiovasc. Res.* 77 302–314 10.1093/cvr/cvm006 18006486

[B39] Ginsburg K. S. Bers D. M. (2004). Modulation of excitation-contraction coupling by isoproterenol in cardiomyocytes with controlled SR Ca^2+^ load and Ca^2+^ current trigger. *J. Physiol.* 556 463–480 10.1113/jphysiol.2003.055384 14724205 PMC1664945

[B40] Grimm M. Brown J. H. (2010). Beta-adrenergic receptor signaling in the heart: role of CaMKII. *J. Mol. Cell. Cardiol.* 48 322–330 10.1016/j.yjmcc.2009.10.016 19883653 PMC2896283

[B41] Guo T. Cornea R. L. Huke S. Camors E. Yang Y. Picht E. (2010). Kinetics of FKBP12.6 binding to ryanodine receptors in permeabilized cardiac myocytes and effects on Ca sparks. *Circ. Res.* 106 1743–1752 10.1161/CIRCRESAHA.110.219816 20431056 PMC2895429

[B42] Guo T. Zhang T. Mestril R. Bers D. M. (2006). Ca^2+^/Calmodulin-dependent protein kinase II phosphorylation of ryanodine receptor does affect calcium sparks in mouse ventricular myocytes. *Circ. Res.* 99 398–406 10.1161/01.RES.0000236756.06252.13 16840718

[B43] Gyorke I. Hester N. Jones L. R. Gyorke S. (2004). The role of calsequestrin, triadin, and junctin in conferring cardiac ryanodine receptor responsiveness to luminal calcium. *Biophys. J.* 86 2121–2128 10.1016/S0006-3495(04)74271-X 15041652 PMC1304063

[B44] Gyorke S. Fill M. (1993). Ryanodine receptor adaptation: control mechanism of Ca(2+)-induced Ca^2+^ release in heart. *Science* 260 807–809 10.1126/science.8387229 8387229

[B45] Hain J. Onoue H. Mayrleitner M. Fleischer S. Schindler H. (1995). Phosphorylation modulates the function of the calcium release channel of sarcoplasmic reticulum from cardiac muscle. *J. Biol. Chem.* 270 2074–2081 10.1074/jbc.270.5.2074 7836435

[B46] Hamilton S. L. Serysheva I. I. (2009). Ryanodine receptor structure: progress and challenges. *J. Biol. Chem.* 284 4047–4051 10.1074/jbc.R800054200 18927076 PMC3837402

[B47] Huke S. Bers D. M. (2008). Ryanodine receptor phosphorylation at Serine 2030, 2808 and 2814 in rat cardiomyocytes. *Biochem. Biophys. Res. Commun.* 376 80–85 10.1016/j.bbrc.2008.08.084 18755143 PMC2581610

[B48] Huttlin E. L. Jedrychowski M. P. Elias J. E. Goswami T. Rad R. Beausoleil S. A. (2010). A tissue-specific atlas of mouse protein phosphorylation and expression. *Cell* 143 1174–1189 10.1016/j.cell.2010.12.001 21183079 PMC3035969

[B49] Ibarra C. Vicencio J. M. Estrada M. Lin Y. Rocco P. Rebellato P. (2013). Local control of nuclear calcium signaling in cardiac myocytes by perinuclear microdomains of sarcolemmal insulin-like growth factor 1 receptors. *Circ. Res.* 112 236–245 10.1161/CIRCRESAHA.112.273839 23118311

[B50] Jiang M. T. Lokuta A. J. Farrell E. F. Wolff M. R. Haworth R. A. Valdivia H. H. (2002). Abnormal Ca^2+^ release, but normal ryanodine receptors, in canine and human heart failure. *Circ. Res.* 91 1015–1022 10.1161/01.RES.0000043663.08689.05 12456487

[B51] Ju Y. K. Liu J. Lee B. H. Lai D. Woodcock E. A. Lei M. (2011). Distribution and functional role of inositol 1,4,5-trisphosphate receptors in mouse sinoatrial node. *Circ. Res.* 109 848–857 10.1161/CIRCRESAHA.111.243824 21852551

[B52] Kirchhefer U. Schmitz W. Scholz H. Neumann J. (1999). Activity of cAMP-dependent protein kinase and Ca^2+^/calmodulin-dependent protein kinase in failing and nonfailing human hearts. *Cardiovasc. Res.* 42 254–261 10.1016/S0008-6363(98)00296-X 10435018

[B53] Kockskämper J. Zima A. V. Roderick H. L. Pieske B. Blatter L. A. Bootman M. D. (2008). Emerging roles of inositol 1,4,5-trisphosphate signaling in cardiac myocytes. *J. Mol. Cell. Cardiol.* 45 128–147 10.1016/j.yjmcc.2008.05.014 18603259 PMC2654363

[B54] Kohlhaas M. Zhang T. Seidler T. Zibrova D. Dybkova N. Steen A. (2006). Increased sarcoplasmic reticulum calcium leak but unaltered contractility by acute CaMKII overexpression in isolated rabbit cardiac myocytes. *Circ. Res.* 98 235–244 10.1161/01.RES.0000200739.90811.9f 16373600

[B55] Krishna A. Valderrabano M. Palade P. T. Clark J. W. Jr (2013). Rate-dependent Ca^2+^ signalling underlying the force-frequency response in rat ventricular myocytes: a coupled electromechanical modeling study. *Theor. Biol. Med. Model.* 10:54 10.1186/1742-4682-10-54 PMC3848742 24020888

[B56] Kushnir A. Marks A. R. (2010). The ryanodine receptor in cardiac physiology and disease. *Adv. Pharmacol.* 59 1–30 10.1016/S1054-3589(10)59001-X 20933197 PMC3023997

[B57] Lehnart S. E. Wehrens X. H. Reiken S. Warrier S. Belevych A. E. Harvey R. D. (2005). Phosphodiesterase 4D deficiency in the ryanodine-receptor complex promotes heart failure and arrhythmias. *Cell* 123 25–35 10.1016/j.cell.2005.07.030 16213210 PMC2901878

[B58] Li J. Imtiaz M. S. Beard N. A. Dulhunty A. F. Thorne R. Vanhelden D. F. (2013). ss-Adrenergic stimulation increases RyR2 activity via intracellular Ca^2+^ and Mg^2+^ regulation. *PLoS ONE* 8:e58334 10.1371/journal.pone.0058334 PMC3606165 23533585

[B59] Li X. Zima A. V. Sheikh F. Blatter L. A. Chen J. (2005). Endothelin-1-induced arrhythmogenic Ca^2+^ signaling is abolished in atrial myocytes of inositol-1,4,5-trisphosphate(IP3)-receptor type 2-deficient mice. *Circ. Res.* 96 1274–1281 10.1161/01.RES.0000172556.05576.4c 15933266

[B60] Li Y. Kranias E. G. Mignery G. A. Bers D. M. (2002). Protein kinase A phosphorylation of the ryanodine receptor does not affect calcium sparks in mouse ventricular myocytes. *Circ. Res.* 90 309–316 10.1161/hh0302.105660 11861420

[B61] Lipp P. Laine M. Tovey S. C. Burrell K. M. Berridge M. J. Li W. (2000). Functional InsP3 receptors that may modulate excitation-contraction coupling in the heart. *Curr. Biol.* 10 939–942 10.1016/S0960-9822(00)00624-2 10959844

[B62] Lokuta A. J. Rogers T. B. Lederer W. J. Valdivia H. H. (1995). Modulation of cardiac ryanodine receptors of swine and rabbit by a phosphorylation-dephosphorylation mechanism. *J. Physiol.* 487 609–622 8544125 10.1113/jphysiol.1995.sp020904 PMC1156649

[B63] Luczak E. D. Anderson M. E. (2014). CaMKII oxidative activation and the pathogenesis of cardiac disease. *J. Mol. Cell. Cardiol*. 10.1016/j.yjmcc.2014.02.004 [Epub ahead of print] PMC4048820 24530899

[B64] Luo D. Yang D. Lan X. Li K. Li X. Chen J. (2008). Nuclear Ca^2+^ sparks and waves mediated by inositol 1,4,5-trisphosphate receptors in neonatal rat cardiomyocytes. *Cell Calcium* 43 165–174 10.1016/j.ceca.2007.04.017 17583790 PMC2266086

[B65] Luo M. Anderson M. E. (2013). Mechanisms of altered Ca(2)(+) handling in heart failure. *Circ. Res.* 113 690–708 10.1161/CIRCRESAHA.113.301651 23989713 PMC4080816

[B66] MacDonnell S. M. Garcia-Rivas G. Scherman J. A. Kubo H. Chen X. Valdivia H. (2008). Adrenergic regulation of cardiac contractility does not involve phosphorylation of the cardiac ryanodine receptor at serine 2808. *Circ. Res.* 102 e65–e72 10.1161/CIRCRESAHA.108.174722 18388322 PMC2652487

[B67] Mackenzie L. Bootman M. D. Laine M. Berridge M. J. Thuring J. Holmes A. (2002). The role of inositol 1,4,5-trisphosphate receptors in Ca(2+) signalling and the generation of arrhythmias in rat atrial myocytes. *J. Physiol.* 541 395–409 10.1113/jphysiol.2001.013411 12042347 PMC2290330

[B68] Maier L. S. Zhang T. Chen L. Desantiago J. Brown J. H. Bers D. M. (2003). Transgenic CaMKIIdeltaC overexpression uniquely alters cardiac myocyte Ca^2+^ handling: reduced SR Ca^2+^ load and activated SR Ca^2+^ release. *Circ. Res.* 92 904–911 10.1161/01.RES.0000069685.20258.F1 12676813

[B69] Marx S. O. Reiken S. Hisamatsu Y. Jayaraman T. Burkhoff D. Rosemblit N. (2000). PKA phosphorylation dissociates FKBP12.6 from the calcium release channel (ryanodine receptor): defective regulation in failing hearts. *Cell* 101 365–376 10.1016/S0092-8674(00)80847-8 10830164

[B70] Matifat F. Hague F. Brule G. Collin T. (2001). Regulation of InsP3-mediated Ca^2+^ release by CaMKII in *Xenopus* oocytes. *Pflugers Arch.* 441 796–801 10.1007/s004240000479 11316263

[B71] Maxwell J. T. Natesan S. Mignery G. A. (2012). Modulation of inositol 1,4,5-trisphosphate receptor type 2 channel activity by Ca^2+^/calmodulin-dependent protein kinase II (CaMKII)-mediated phosphorylation. *J. Biol. Chem.* 287 39419–39428 10.1074/jbc.M112.374058 23019322 PMC3501088

[B72] Meissner G. (2004). Molecular regulation of cardiac ryanodine receptor ion channel. *Cell Calcium* 35 621–628 10.1016/j.ceca.2004.01.015 15110152

[B73] Mignery G. A. Newton C. L. Archer B. T. III Sudhof T. C. (1990). Structure and expression of the rat inositol 1,4,5-trisphosphate receptor. *J. Biol. Chem.* 265 12679–12685 2165071

[B74] Mignery G. A. Sudhof T. C. (1990). The ligand binding site and transduction mechanism in the inositol-1,4,5-triphosphate receptor. *EMBO J.* 9 3893–3898 2174351 10.1002/j.1460-2075.1990.tb07609.x PMC552159

[B75] Mohler P. J. Schott J. J. Gramolini A. O. Dilly K. W. Guatimosim S. Dubell W. H. (2003). Ankyrin-B mutation causes type 4 long-QT cardiac arrhythmia and sudden cardiac death. *Nature* 421 634–639 10.1038/nature01335 12571597

[B76] Molkentin J. D. (2000). Calcineurin and beyond: cardiac hypertrophic signaling. *Circ. Res.* 87 731–738 10.1161/01.RES.87.9.731 11055975

[B77] Molkentin J. D. Lu J. R. Antos C. L. Markham B. Richardson J. Robbins J. (1998). A calcineurin-dependent transcriptional pathway for cardiac hypertrophy. *Cell* 93 215–228 10.1016/S0092-8674(00)81573-1 9568714 PMC4459646

[B78] Newton C. L. Mignery G. A. Sudhof T. C. (1994). Co-expression in vertebrate tissues and cell lines of multiple inositol 1,4,5-trisphosphate (InsP3) receptors with distinct affinities for InsP3. *J. Biol. Chem.* 269 28613–28619 7961809

[B79] Perez P. J. Ramos-Franco J. Fill M. Mignery G. A. (1997). Identification and functional reconstitution of the type 2 inositol 1,4,5-trisphosphate receptor from ventricular cardiac myocytes. *J. Biol. Chem.* 272 23961–23969 10.1074/jbc.272.38.23961 9295347

[B80] Pietri F. Hilly M. Mauger J. P. (1990). Calcium mediates the interconversion between two states of the liver inositol 1,4,5-trisphosphate receptor. *J. Biol. Chem.* 265 17478–17485 2170381

[B81] Pinna L. A. Ruzzene M. (1996). How do protein kinases recognize their substrates? *Biochim. Biophys. Acta* 1314 191–225 10.1016/S0167-4889(96)00083-3 8982275

[B82] Priori S. G. Chen S. R. (2011). Inherited dysfunction of sarcoplasmic reticulum Ca^2+^ handling and arrhythmogenesis. *Circ. Res.* 108 871–883 10.1161/CIRCRESAHA.110.226845 21454795 PMC3085083

[B83] Purohit A. Rokita A. G. Guan X. Chen B. Koval O. M. Voigt N. (2013). Oxidized Ca(2+)/calmodulin-dependent protein kinase II triggers atrial fibrillation. *Circulation* 128 1748–1757 10.1161/CIRCULATIONAHA.113.003313 24030498 PMC3876034

[B84] Ramos-Franco J. Fill M. Mignery G. A. (1998). Isoform-specific function of single inositol 1,4,5-trisphosphate receptor channels. *Biophys. J.* 75 834–839 10.1016/S0006-3495(98)77572-1 9675184 PMC1299757

[B85] Respress J. L. Van Oort R. J. Li N. Rolim N. Dixit S. S. Dealmeida A. (2012). Role of RyR2 phosphorylation at S2814 during heart failure progression. *Circ. Res.* 110 1474–1483 10.1161/CIRCRESAHA.112.268094 22511749 PMC3371642

[B86] Rodriguez P. Bhogal M. S. Colyer J. (2003). Stoichiometric phosphorylation of cardiac ryanodine receptor on serine 2809 by calmodulin-dependent kinase II and protein kinase A. *J. Biol. Chem.* 278 38593–38600 10.1074/jbc.C301180200 14514795

[B87] Said M. Becerra R. Valverde C. A. Kaetzel M. A. Dedman J. R. Mundina-Weilenmann C. (2011). Calcium-calmodulin dependent protein kinase II (CaMKII): a main signal responsible for early reperfusion arrhythmias. *J. Mol. Cell. Cardiol.* 51 936–944 10.1016/j.yjmcc.2011.08.010 21888910 PMC3208750

[B88] Seiler S. Wegener A. D. Whang D. D. Hathaway D. R. Jones L. R. (1984). High molecular weight proteins in cardiac and skeletal muscle junctional sarcoplasmic reticulum vesicles bind calmodulin, are phosphorylated, and are degraded by Ca^2+^-activated protease. *J. Biol. Chem.* 259 8550–8557 6203912

[B89] Shan J. Betzenhauser M. J. Kushnir A. Reiken S. Meli A. C. Wronska A. (2010a). Role of chronic ryanodine receptor phosphorylation in heart failure and beta-adrenergic receptor blockade in mice. *J. Clin. Invest.* 120 4375–4387 10.1172/JCI37649 21099115 PMC2993577

[B90] Shan J. Kushnir A. Betzenhauser M. J. Reiken S. Li J. Lehnart S. E. (2010b). Phosphorylation of the ryanodine receptor mediates the cardiac fight or flight response in mice. *J. Clin. Invest.* 120 4388–4398 10.1172/JCI32726 21099118 PMC2993575

[B91] Sienaert I. De Smedt H. Parys J. B. Missiaen L. Vanlingen S. Sipma H. (1996). Characterization of a cytosolic and a luminal Ca^2+^ binding site in the type I inositol 1,4,5-trisphosphate receptor. *J. Biol. Chem.* 271 27005–27012 10.1074/jbc.271.43.27005 8900188

[B92] Sienaert I. Missiaen L. De Smedt H. Parys J. B. Sipma H. Casteels R. (1997). Molecular and functional evidence for multiple Ca^2+^-binding domains in the type 1 inositol 1,4,5-trisphosphate receptor. *J. Biol. Chem.* 272 25899–25906 10.1074/jbc.272.41.25899 9325322

[B93] Signore S. Sorrentino A. Ferreira-Martins J. Kannappan R. Shafaie M. Del Ben F. (2013). Inositol 1, 4, 5-trisphosphate receptors and human left ventricular myocytes. *Circulation* 128 1286–1297 10.1161/CIRCULATIONAHA.113.002764 23983250 PMC3873649

[B94] Sossalla S. Fluschnik N. Schotola H. Ort K. R. Neef S. Schulte T. (2010). Inhibition of elevated Ca^2+^/calmodulin-dependent protein kinase II improves contractility in human failing myocardium. *Circ. Res.* 107 1150–1161 10.1161/CIRCRESAHA.110.220418 20814023

[B95] Stange M. Xu L. Balshaw D. Yamaguchi N. Meissner G. (2003). Characterization of recombinant skeletal muscle (Ser-2843) and cardiac muscle (Ser-2809) ryanodine receptor phosphorylation mutants. *J. Biol. Chem.* 278 51693–51702 10.1074/jbc.M310406200 14532276

[B96] Supattapone S. Worley P. F. Baraban J. M. Snyder S. H. (1988). Solubilization, purification, and characterization of an inositol trisphosphate receptor. *J. Biol. Chem.* 263 1530–1534 2826483

[B97] Tadevosyan A. Vaniotis G. Allen B. G. Hebert T. E. Nattel S. (2012). G protein-coupled receptor signalling in the cardiac nuclear membrane: evidence and possible roles in physiological and pathophysiological function. *J. Physiol.* 590 1313–1330 10.1113/jphysiol.2011.222794 22183719 PMC3382322

[B98] Taigen T. De Windt L. J. Lim H. W. Molkentin J. D. (2000). Targeted inhibition of calcineurin prevents agonist-induced cardiomyocyte hypertrophy. *Proc. Natl. Acad. Sci. U.S.A.* 97 1196–1201 10.1073/pnas.97.3.1196 10655507 PMC15566

[B99] Takasago T. Imagawa T. Shigekawa M. (1989). Phosphorylation of the cardiac ryanodine receptor by cAMP-dependent protein kinase. *J. Biochem.* 106 872–877 2613695 10.1093/oxfordjournals.jbchem.a122945

[B100] Takeshima H. Komazaki S. Hirose K. Nishi M. Noda T. Iino M. (1998). Embryonic lethality and abnormal cardiac myocytes in mice lacking ryanodine receptor type 2. *EMBO J.* 17 3309–3316 10.1093/emboj/17.12.3309 9628868 PMC1170669

[B101] Taylor C. W. Tovey S. C. (2010). IP(3) receptors: toward understanding their activation. *Cold Spring Harb. Perspect. Biol.* 2:a004010 10.1101/cshperspect.a004010 PMC2982166 20980441

[B102] Terentyev D. Viatchenko-Karpinski S. Gyorke I. Terentyeva R. Gyorke S. (2003). Protein phosphatases decrease sarcoplasmic reticulum calcium content by stimulating calcium release in cardiac myocytes. *J. Physiol.* 552 109–118 10.1113/jphysiol.2003.046367 12897175 PMC2343319

[B103] Timerman A. P. Onoue H. Xin H. B. Barg S. Copello J. Wiederrecht G. (1996). Selective binding of FKBP12.6 by the cardiac ryanodine receptor. *J. Biol. Chem.* 271 20385–20391 10.1074/jbc.271.34.20385 8702774

[B104] Uchida K. Miyauchi H. Furuichi T. Michikawa T. Mikoshiba K. (2003). Critical regions for activation gating of the inositol 1,4,5-trisphosphate receptor. *J. Biol. Chem.* 278 16551–16560 10.1074/jbc.M300646200 12621039

[B105] Uehara A. Yasukochi M. Mejia-Alvarez R. Fill M. Imanaga I. (2002). Gating kinetics and ligand sensitivity modified by phosphorylation of cardiac ryanodine receptors. *Pflugers Arch.* 444 202–212 10.1007/s00424-002-0791-3 11976933

[B106] Valdivia H. H. (2012). Ryanodine receptor phosphorylation and heart failure: phasing out s2808 and ``criminalizing'' s2814. *Circ. Res.* 110 1398–1402 10.1161/CIRCRESAHA.112.270876 22628571 PMC3386797

[B107] Valdivia H. H. (2013). ``Ionic basis of sarcoplasmic reticulum ion fluxes in heart muscle,'' in *Cardiac Electrophysiology: From Cell to Bedside* 5th Edn eds Zipes D. Jalife J. (New York: Saunders)

[B108] Valdivia H. H. Kaplan J. H. Ellis-Davies G. C. Lederer W. J. (1995). Rapid adaptation of cardiac ryanodine receptors: modulation by Mg^2+^ and phosphorylation. *Science* 267 1997–2000 10.1126/science.7701323 7701323 PMC4242209

[B109] Vanderheyden V. Devogelaere B. Missiaen L. De Smedt H. Bultynck G. Parys J. B. (2009). Regulation of inositol 1,4,5-trisphosphate-induced Ca^2+^ release by reversible phosphorylation and dephosphorylation. *Biochim. Biophys. Acta* 1793 959–970 10.1016/j.bbamcr.2008.12.003 19133301 PMC2693466

[B110] van Oort R. J. Mccauley M. D. Dixit S. S. Pereira L. Yang Y. Respress J. L. (2010). Ryanodine receptor phosphorylation by calcium/calmodulin-dependent protein kinase II promotes life-threatening ventricular arrhythmias in mice with heart failure. *Circulation* 122 2669–2679 10.1161/CIRCULATIONAHA.110.982298 21098440 PMC3075419

[B111] Van Petegem F. (2012). Ryanodine receptors: structure and function. *J. Biol. Chem.* 287 31624–31632 10.1074/jbc.R112.349068 22822064 PMC3442496

[B112] Walaas S. I. Horn R. S. Albert K. A. Adler A. Walaas O. (1988). Phosphorylation of multiple sites in a 15,000 dalton proteolipid from rat skeletal muscle sarcolemma, catalyzed by adenosine 3′, 5′-monophosphate-dependent and calcium/phospholipid-dependent protein kinases. *Biochim. Biophys. Acta* 968 127–137 10.1016/0167-4889(88)90052-3 3337842

[B113] Wehrens X. H. Lehnart S. E. Reiken S. R. Marks A. R. (2004). Ca^2+^/calmodulin-dependent protein kinase II phosphorylation regulates the cardiac ryanodine receptor. *Circ. Res.* 94 e61–e70 10.1161/01.RES.0000125626.33738.E2 15016728

[B114] Wehrens X. H. Lehnart S. E. Reiken S. Vest J. A. Wronska A. Marks A. R. (2006). Ryanodine receptor/calcium release channel PKA phosphorylation: a critical mediator of heart failure progression. *Proc. Natl. Acad. Sci. U.S.A.* 103 511–518 10.1073/pnas.0510113103 16407108 PMC1334677

[B115] Witcher D. R. Kovacs R. J. Schulman H. Cefali D. C. Jones L. R. (1991). Unique phosphorylation site on the cardiac ryanodine receptor regulates calcium channel activity. *J. Biol. Chem.* 266 11144–11152 1645727

[B116] Wu X. Zhang T. Bossuyt J. Li X. Mckinsey T. A. Dedman J. R. (2006). Local InsP3-dependent perinuclear Ca^2+^ signaling in cardiac myocyte excitation-transcription coupling. *J. Clin. Invest.* 116 675–682 10.1172/JCI27374 16511602 PMC1386110

[B117] Wu Y. Temple J. Zhang R. Dzhura I. Zhang W. Trimble R. (2002). Calmodulin kinase II and arrhythmias in a mouse model of cardiac hypertrophy. *Circulation* 106 1288–1293 10.1161/01.CIR.0000027583.73268.E7 12208807

[B118] Xiao B. Jiang M. T. Zhao M. Yang D. Sutherland C. Lai F. A. (2005). Characterization of a novel PKA phosphorylation site, serine-2030, reveals no PKA hyperphosphorylation of the cardiac ryanodine receptor in canine heart failure. *Circ. Res.* 96 847–855 10.1161/01.RES.0000163276.26083.e8 15790957

[B119] Xiao B. Sutherland C. Walsh M. P. Chen S. R. (2004). Protein kinase A phosphorylation at serine-2808 of the cardiac Ca^2+^-release channel (ryanodine receptor) does not dissociate 12.6-kDa FK506-binding protein (FKBP12.6). *Circ. Res.* 94 487–495 10.1161/01.RES.0000115945.89741.22 14715536

[B120] Xiao B. Zhong G. Obayashi M. Yang D. Chen K. Walsh M. P. (2006). Ser-2030, but not Ser-2808, is the major phosphorylation site in cardiac ryanodine receptors responding to protein kinase A activation upon beta-adrenergic stimulation in normal and failing hearts. *Biochem. J.* 396 7–16 10.1042/BJ20060116 16483256 PMC1449991

[B121] Xiao J. Tian X. Jones P. P. Bolstad J. Kong H. Wang R. (2007). Removal of FKBP12.6 does not alter the conductance and activation of the cardiac ryanodine receptor or the susceptibility to stress-induced ventricular arrhythmias. *J. Biol. Chem.* 282 34828–34838 10.1074/jbc.M707423200 17921453 PMC2760432

[B122] Yang D. Zhu W. Z. Xiao B. Brochet D. X. Chen S. R. Lakatta E. G. (2007). Ca^2+^/calmodulin kinase II-dependent phosphorylation of ryanodine receptors suppresses Ca^2+^ sparks and Ca^2+^ waves in cardiac myocytes. *Circ. Res.* 100 399–407 10.1161/01.RES.0000258022.13090.55 17234969

[B123] Yang Y. Guo T. Oda T. Chakraborty A. Chen L. Uchinoumi H. (2014). Cardiac myocyte Z-line calmodulin is mainly RyR2-bound, and reduction is arrhythmogenic and occurs in heart failure. *Circ. Res.* 114 295–306 10.1161/CIRCRESAHA.114.302857 24186966 PMC4004530

[B124] Yuchi Z. Lau K Van Petegem F. (2012). Disease mutations in the ryanodine receptor central region: crystal structures of a phosphorylation hot spot domain. *Structure* 20 1201–1211 10.1016/j.str.2012.04.015 22705209

[B125] Zhang B. X. Zhao H. Muallem S. (1993). Ca(2+)-dependent kinase and phosphatase control inositol 1,4,5-trisphosphate-mediated Ca^2+^ release. Modification by agonist stimulation. *J. Biol. Chem.* 268 10997–11001 8388379

[B126] Zhang H. Makarewich C. A. Kubo H. Wang W. Duran J. M. Li Y. (2012). Hyperphosphorylation of the cardiac ryanodine receptor at serine 2808 is not involved in cardiac dysfunction after myocardial infarction. *Circ. Res.* 110 831–840 10.1161/CIRCRESAHA.111.255158 22302785 PMC3322671

[B127] Zhu D. M. Tekle E. Chock P. B. Huang C. Y. (1996). Reversible phosphorylation as a controlling factor for sustaining calcium oscillations in HeLa cells: involvement of calmodulin-dependent kinase II and a calyculin A-inhibitable phosphatase. *Biochemistry* 35 7214–7223 10.1021/bi952471h 8679550

[B128] Zhu W. Zou Y. Shiojima I. Kudoh S. Aikawa R. Hayashi D. (2000). Ca^2+^/calmodulin-dependent kinase II and calcineurin play critical roles in endothelin-1-induced cardiomyocyte hypertrophy. *J. Biol. Chem.* 275 15239–15245 10.1074/jbc.275.20.15239 10809760

[B129] Zima A. V. Blatter L. A. (2004). Inositol-1,4,5-trisphosphate-dependent Ca(2+) signalling in cat atrial excitation-contraction coupling and arrhythmias. *J. Physiol.* 555 607–615 10.1113/jphysiol.2003.058529 14754996 PMC1664857

[B130] Zucchi R. Ronca-Testoni S. (1997). The sarcoplasmic reticulum Ca^2+^ channel/ryanodine receptor: modulation by endogenous effectors, drugs and disease states. *Pharmacol. Rev.* 49 1–51 9085308

